# Residents’ and supervisors’ experiences when using a feedback-model in post-graduate medical education

**DOI:** 10.1186/s12909-022-03969-5

**Published:** 2022-12-23

**Authors:** Martin Lägervik, Karin Thörne, Sofi Fristedt, Maria Henricson, Berith Hedberg

**Affiliations:** 1 Jönköping Academy, Jönköping University, 551 11 Jönköping, Sweden; 2 Futurum – the Academy for Health and Care, Region Jönköping County, 551 11 Jönköping, Sweden; 3 Department of Health Sciences, Faculty of Medicine, Lund University, 221 00 Lund, Sweden; 4 Department of Caring Sciences, University of Borås, 501 90 Borås, Sweden

**Keywords:** Feedback, Supervision, Post-graduate medical education (PGME), Supervisor, Resident, Feedback model, Learning environment

## Abstract

**Background:**

Supervisors play a key part as role models and supporting the learning during residents’ post-graduate medical education, but sometimes lack sufficient pedagogic training and are challenged by high demands in today’s healthcare. The aim of this study was to describe the strengths and areas for improvement identified in the supervision process by residents and supervisors in post-graduate medical education.

**Methods:**

This study included supervisors and residents working at departments and health centres who have used a web-based questionnaire, as a part of the Evaluation and Feedback For Effective Clinical Teaching (EFFECT) model, during the period 2016–2019. Descriptive statistics and content analysis were used to analyse ratings and comments to describe strengths and areas for improvement in the supervision process.

**Results:**

The study included 287 resident evaluations of supervisors and 78 self-evaluations by supervisors. The supervisor as a role model, being available, and, giving personal support, were the three most important strengths identified by the residents and supervisors. Residents in primary care also identified the role modelling of general practice competence as a strength, whereas residents and supervisors in hospital departments addressed supervisors as energetic and showing work was fun. The area with the need of most improvement was, Giving and receiving feedback.

**Conclusions:**

To be able to give feedback, residents and supervisors, needed to see each other in work, and the learning environment had to offer time and space to pedagogical processes, like feedback, to improve the learning environment.

## Background

Post-graduate medical education (PGME) is based on learning in the workplace, which stimulates the residents’ motivation to learn and offers great possibilities for interactions between residents and supervisors [1]. The learning environment should provide tasks relevant for learning with support and supervision from supervisors where residents can improve their skills and progress, in a safe context, without fear of being judged [2, 3]. Inspiring learning environments are associated with competent supervisors [4], who are aware of what and how to supervise [5]. Supervision is a two-way process between supervisors and residents, which is shaped by both and in need of support from more experienced colleagues and residents agency [6]. Giving feedback, defined as a process in which supervisors identify the similarities and differences between the current and desirable performance, to help the learner develop their competence [2, 7]. Feedback has been shown to be more effective, if it is well-founded and received in time [8]. Feedback in the other direction, up-ward feedback, from residents to supervisors, is often challenging and less researched [8]. Here, the residents need to understand their own role as mediators of feedback, have a language to provide it and work in a learning environment where feedback is requested [9, 10].

However, potentially threatening the learning environment, supervisors report a lack of formal education and are not always aware of how to supervise and how to assess residents [11, 12]. Another challenge every day is to balance role modelling and handling heavy workload as well as demands from patients [13, 14].

There is a need for instruments and models for developing and strengthening supervisors [15–17], as supervisors who get feedback improve their supervision [18]. The Evaluation and Feedback For Effective Clinical Teaching (EFFECT) is a feedback model developed in the Netherlands. The EFFECT model includes CanMEDS’ definition of the physicians’ seven core competencies: medical expert, communicator, collaborator, scholar, manager, health advocate and professional [19]. Research has shown that the EFFECT model contributes to improved feedback between residents and supervisors as well as stimulating the learning environment [20]. Since 2015, the model has been implemented in a regional county council in Sweden, in close collaboration with the inventor.

Because the EFFECT model has been used at hospital departments, rather than in primary care, it is also important to generate knowledge from the latter context. Moreover, giving and receiving feedback is not always easy and the EFFECT model approach feedback in the upward direction i.e., from resident to supervisor. This can challenge hierarchy and less is known about how this is experienced, as well as how it affects both parties. Moreover, it is important to establish knowledge on new tools, such as EFFECT and as far as we know, there are no similar models used in Sweden. The aim of this study was to describe the strengths and areas for improvement identified in the supervision process by residents and supervisors in Swedish PGME, in hospital departments and within primary care.

## Methods

The present study combined quantitative and qualitative design [21]. This study is part of a larger PhD project concerning upward feedback, where other steps and outcomes generated by the EFFECT model will be analysed in further research.

The study took place in a region in the south of Sweden, with 365 000 citizens, supported by three hospitals and fifty health centres in primary care. In the region, there are 365 residents of which 125 work in primary care, and 765 physicians including 180 general practitioners working in primary care. Departments and health centres employ the residents, which means that they work as colleagues. Every medical speciality has their own program director to support the residents, the supervisors, and the operational managers at departments/health centres. The main supervisors must have undergone supervisor training, but all physicians supervise at departments and in primary care. All PGME programmes are outcome-based lasting at least five years.

### Data collection and participants

The EFFECT model has five steps where the central part is the feedback dialogue where two residents, with the support of a neutral moderator, give feedback to one supervisor. As a basis before the dialogue, residents, and supervisors answer the EFFECT questionnaire, covering a range of aspects of supervision. The supervisors performed self-evaluations and residents evaluated as many as possible of the supervisors at their department or health centre. The results of the EFFECT questionnaires are compiled into a report that forms the basis when residents give feedback to their supervisors.

The EFFECT questionnaire was web-based and had 58 questions divided in seven domains (see Table 1). The EFFECT questionnaire has been validated in the Netherlands and Lithuania [22, 23] and was translated into Swedish in line with standardised procedures [24]. The original EFFECT questionnaire included 58 questions but in 2018 there was a revision in the Netherlands, where three questions where deleted, as they did not provide new information.

**Table 1:** The EFFECT questionnaire. Domains, number of questions and examples

Domains	Number of questions	Examples of questions addressing residents
Role modelling	15	By observing my supervisor, I learn how to treat patient respectfully
Task assignment	6 (5)^a^	My supervisor gives me tasks that suit my current level of training
Planning	3	My supervisor is available when I need him/her during my shift
Feedback	12	My supervisor discusses what I can improve
Teaching methodology	8 (7)^a^	My supervisor stimulates me to find out things for myself
Personal support	6 (5)^a^	My supervisor treats me respectfully
Assessment	8	My supervisor reviews my portfolio during the assessment
Total	58 (55)^a^	

^a^Number of questions after review 2018

All questions were rated using a six-point Likert scale: 1) Critical, can’t continue, 2) Insufficient, big improvements needed, 3) Mediocre, improvements needed, 4) Good, some details can be improved, 5) Very good, continue the good work, 6) Excellent, an example to everyone. There was also a non-response alternative (cannot be answered). As part of the questionnaire, the residents and supervisors also responded to open-ended questions about the strengths of the supervisor, areas for improvements and specific tips or suggestions.

The implementation of the EFFECT model started in 2016 with a pilot including one department and four health centres, where program directors and operational managers showed interest. The EFFECT questionnaire was distributed through a web-based system and available for answering within four weeks, with a reminder issued to participants after two weeks. Five departments and nine health centres, who had participated in the EFFECT model, during the period 2016–2019, were included (see Table 2).

**Table 2:** The study population and number of questionnaires

Hospital departments	Supervisors (n)	Supervisors' self-evaluations (n)	Residents (n)	Residents’ evaluations (n)
Department 1, 2016 (pilot)	9	7	5	28
Department 2, 2017	18	18	14	70
Department 3, 2018	2	2	2	4
Department 4, 2018*	16	12	10	80
Department 5, 2019*	13	9	4	42
**Total**	58	48	35	224
**Primary care**	**Supervisors (n)**	**Supervisors’ self-evaluations (n)**	**Residents (n)**	**Residents’ evaluations (n)**
Health centres 1–4, 2016 (pilot)	14	11	12	21
Health centre 5, 2017	8	4	5	8
Health centre 6, 2018	5	5	4	20
Health centre 7, 2018*	7	5	7	5
Health centre 8, 2018*	3	2	4	6
Health centre 9, 2018*	6	3	7	3
**Total**	43	30	39	63

### Data analysis

The EFFECT questionnaire data was analysed as suggested, i.e., median for data from residents and supervisors, in hospital departments and primary care, as well as separately for each domain in the EFFECT questionnaire [25].

The open-ended questions in the questionnaires, were analysed using summative content analysis [26]. All comments from residents and supervisors in departments and health centres were read through line-by-line and coded by ML, KT, MH and BH, whereby meaning units were created and categorized. To increase credibility of the categories, all authors read all meaning units and categorized them, resulting in a joint assessment of the categories. The meaning units within the categories were read through again and the first author formed sub-categories. To respond to the aim of this study, the categories were allocated into the concept of the seven domains in the EFFECT questionnaire as a deductive approach to content analysis [27].

### Ethics

The Research Committee of Ethics at the School of Health and Welfare, Jönköping University has reviewed the project without any comments. Residents and supervisors received oral and written information about the EFFECT model and taking part was voluntary. The material was anonymised, and personal information cannot be traced whereby participants gain confidentiality [28, 29].

## Results

The ratings in the EFFECT questionnaire are presented with median and the responses to open-ended questions displayed in structure of the categories relating to the seven domains in the EFFECT questionnaire.

### Quantitative results from the questionnaires

Residents, in departments and primary care, rated their supervisors in the same way, while the supervisors’ self-ratings differed between departments and primary care. Based on calculated median for ratings, the domains are sorted for supervisors and residents, in hospital departments as well as primary care (Table 3). The Table 3 also report each groups response rates per domain. Supervisors in departments and primary care have answered most questions, response rate 89% or more, while residents have answered to a lower degree, 60–90%. The lowest response rate was in domain Feedback, 60% and 68%, residents in hospital departments and primary care, respectively. Only main supervisors have been able to answer questions in domain Assessment, following only residents connected to that supervisor could answer the questions in the domain. This explains the fewer answers in the Assessment domain.

**Table 3:** The result of the quantitative analysis presented with median including response rates (rr) in percent, for supervisors and residents in hospital departments and primary care, separately

Scale (median)		Hospital departments		Primary care	
		Supervisors (rr)	Residents (rr)	Supervisors (rr)	Residents (rr)
Excellent, an example to everyone	(6)		Personal support (84%)		Personal support (87%)
Very good, continue the good work	(5)	Role modelling (98%) Personal support (97%) Task assignment (96%)	Assessment (87%) ^a^(*n* = 34) Task assignment (86%) Role modelling (83%) Planning (79%) Feedback (60%)	Planning (98%) Task assignment (97%) Personal support (96%)	Planning (90%) Assessment (89%) ^a^(*n* = 20) Role modelling (75%) Task assignment (74%) Feedback (68%)
Good, with some things to improve	(4)	Assessment (98%) *(*n* = 32) Planning (96%) Feedback (96%) Teaching Methodology (96%)		Teaching Methodology (96%) Role modelling (92%) Feedback (91%)	
Mediocre, improvements needed	(3)			Assessment (89%) ^a^(*n* = 18)	
Insufficient, big improvements needed	(2)				
Critical, can’t continue	(1)				

^a^*n* = number of questionnaires in domain Assessment

### Qualitative results of the open-ended questions in the questionnaires

The analysis of the open-ended question findings is presented for each category and allocated to the seven domains in the EFFECT questionnaire (see Table 4). The domain that obtained the most meaning units is presented first and the domain with the least is presented last. There were 755 meaning units referred to as strengths and 288 meaning units marked as areas for improvement (1043 in total). Many comments were short such as this comment from a resident in a department about a supervisor’s strengths: “available, engaged, competent, honest, encouraging”. There were more than three times as many evaluations from residents in departments (*n* = 224) than in primary care (*n* = 63), and consequently more comments from them, although residents in primary care (*n* = 39) were slightly numerous than in departments (*n* = 35) (see Table 2).

**Table 4:** The categories in the qualitative analysis allocated to the seven domains in the EFFECT questionnaire, presented with number of meaning units (n) and showing the relation between strengths and areas for improvement

Domain (n)		Categories (n)		Strengths (n)	Areas for improvement (n)
Role modelling	(329)	**Medical and scientific competence**	120	113	7
		**Personal qualities**	116	66	50
		**Patient care qualities**	69	44	25
		**General practice competence**	24	24	0
Personal support	(274)	**Kind and empathetic**	73	65	8
		**Supportive**	71	70	1
		**Energizer**	68	66	2
		**Calm and safe**	62	62	0
Planning	(158)	**Available in time**	115	88	27
		**Mentally available**	26	17	9
		**Scheduling**	19	0	19
Feedback	(105)	**Giving and receiving feedback**	98	34	64
		**Structured feedback**	7	1	6
Task assignment	(87)	**Giving resident space**	61	50	11
		**Challenge residents**	24	12	12
Teaching methodology	(84)	**Pedagogical qualities**	59	41	18
		**Connection to curriculum**	25	2	23
Assessment	(6)	**Formal assessment**	4	0	4
		**Planning PGME**	2	0	2

### Role modelling

Four categories were identified as part of the domain Role modelling, i.e., Medical and scientific competence, Personal support, Patient qualities, and, General practice competence, were identified.

#### Medical and scientific competence

Most comments were seen in the category, Medical and scientific competence, mainly categorised as strengths. Supervisors were seen as being excellent in their medical field, scientifically skilled, having long experience and being up to date. A resident in a department wrote: *“Very medically competent and a role-model with patients!”* Areas for improvement concerned more scientific knowledge and connection to evidence-based medicine.

#### Personal qualities

Three sub-categories were identified: Being clear, painstaking, and structured, Self-esteem and self-reflection, and Balance work/spare time. In the first sub-category, Being clear painstaking, and structured, residents and supervisors described how it is important to be clear about how to give and receive information and perform tasks. The absence of clarity contributed to ambiguity among residents if their supervisors were satisfied with their performances or not. Regarding their supervisors, a resident in a department wrote: *“…could be clearer as physician on-call”* and from a resident in primary care: *“…can be more structured when giving feedback and connect to curriculum”.* In the second sub-category, Self-esteem and self-reflection, residents commented about supervisors to believe in themselves and to be more open about their thoughts: *“She does not know how good she is”*, as a resident in a department wrote. Only a few comments were dealing with the third sub-category, Balance work/spare time, equally as strengths and areas for improvements. Residents appreciated talking about how to balance work and spare time as well as handle stressful situations, but conversations about those issues could increase. A resident in primary care wrote: *“More discussions about work and spare-time”.*

#### Patient care qualities

Residents and supervisors pointed out strengths like communication with patients, collaboration with other health professionals and the importance of being aware of role modelling. A resident in a department wrote about the supervisor: *“…is a role model when it comes to talking to children and their parents”,* and a resident in primary care: “…*is a good role model in how to work as a specialist in general medicine*”. The areas for improvement concerned dealing with difficult situations and overall being a role model but mostly about communication skills, like a resident in a department wrote: *“[my supervisor] could be clearer in conversations with patients.*”

#### General practice competence

Residents in primary care identified the category, General practice competence, as the main strength together with medical and scientific competence, i.e., a resident in primary care wrote *“You are well experienced in general practice and you teach me about that”.* It was not commented by the supervisors and there was no direct question about the competence in the EFFECT questionnaire.

### Personal support

The four categories, Kind and empathetic*,* Supportive*,* Energizer, and, Calm and safe, were allocated to the domain Personal support. The categories included mainly strengths and few areas for improvement.

#### Kind and emphatic

Supervisors were described as empathetic, respectful, and kind, by the residents, as well as patient and reflecting. A supervisor in primary care wrote: *“[I am] listening and take time when the resident needs me”.*

#### Supportive

The category refers to supervisors being available in time and on call, helping residents with medical tasks as well as personal problems, i.e., as a resident in a department wrote: *“[my supervisor] gives me good support in difficult situations*”.

#### Energizer

The category was mainly commented by residents and supervisors in departments. Supervisors wrote that they wanted to show that work is fun, and residents commented about getting energy and feeling encouraged by their supervisors. A supervisor in a department wrote: *“[I am] enthusiastic, I want to show you need to have fun at work”,* and another supervisor in a department commented about observing progress: *“It is incredible to see residents grow.”*

#### Calm and safe

Both residents and supervisors described the category as a strength. Many residents described their supervisors as calm, with the ability to handle stress, and all kinds of questions and situations. A resident in primary care wrote: *“[my supervisor is] very calm and I know I can bring my problems to [supervisor] without worries.”*

### Planning

The three categories: Available in time, Mentally available and Scheduling, were allocated to the domain Planning.

#### Available in time

To be available for questions and support when needed were important to both residents and supervisors. A resident in primary care wrote: *“[my supervisor is] always calm and takes time, regardless of how stressful the situation is”* which was a recurrent theme. Areas for improvement were situations where supervisors were not available according to workload or not working together. A supervisor in a department commented: *“Time, time, time, I have too many tasks to be able to supervise and reflect upon it*”.

#### Mentally available

Residents and supervisors commented about the importance of supervisors being open for discussions and being mentally available. In the departments, residents asked for more support when working at the emergency unit as an area for improvement, i.e., a resident in a department wrote: *“I am not sure if [my supervisor] is satisfied with my medical decisions.”*

#### Scheduling

The category was an area for improvement with comments, mainly from departments about the need for more planned and structured supervision time. A supervisor in a department wrote: *“I am not good at following the residency plan and I should book more formal supervision times”.*

### Feedback

Two categories were identified and allocated to the domain Feedback: Giving and receiving feedback, and, Structured feedback.

#### Giving and receiving feedback

Giving and receiving feedback received the most improvement comments. Residents asked for more feedback, both positive and constructive, and supervisors wrote they should give more feedback, both direct and more structured. A resident in a department wrote: *“Please, give me more constructive feedback”* and a supervisor in departments commented: *“I have to improve giving positive and negative feedback.”*

#### Structured feedback

There were few comments about giving or receiving structured feedback and **i**t was mainly an area for improvement. Residents and supervisors suggested formal assessments like sit-ins, where the supervisor observes a patient visit to the resident and gives structured feedback afterwards. Residents and supervisors also asked for the use of other instruments that generates structured feedback, in daily work and at supervision time. A resident in primary care wrote: *“…would be good with more structured feedback, preferably using special instrument for this”.*

### Task assignment

The two categories, Giving residents space and Challenging residents, were allocated to the domain Task assignment.

#### Giving resident space

Both residents and supervisors described the value of giving space for residents in daily work, taking care of patients independently and progress in medical tasks, as well as discussing patients where supervisors acted as a sounding board. A supervisor in a department wrote: *“…my strongest points are about lifting and strengthen the individual, as well as give space to discuss questions around leadership, collaboration, difficult decisions *etc*.”* The areas for improvement concerned supervisors not inviting residents to take part in medical tasks, as a resident in a department wrote: *“[my supervisor] could let me handle more difficult patients with supervision.”*

#### Challenging residents

The category were almost only commented in departments, both from residents and supervisors, equally as strengths and areas for improvement. Residents described how supervisors challenged them in daily work and being ready to step-in when needed, but also the need for more questions and demands to be able to develop. A resident in a department wrote: *“You are good in questioning and making me as a resident think through and explain my assessments”.*

### Teaching methodology

The categories, Pedagogical qualities, and, Connection to curriculum, were allocated to the domain Teaching methodology.

#### Pedagogical qualities

The comments in the category were about supervisors being pedagogical and inspiring teachers, as well as being interested in supervision. A resident in a department wrote: *“[my supervisor] give well-sought-out treatment advice and theoretical backgrounds”.* The pedagogical skills were also areas for improvement, where residents asked for more guidance rather than lectures, as a resident in a department wrote: *“…long discussions without decisions…”*.

#### Connection to curriculum

The category was mostly an area for improvement, where residents and supervisors wrote they wanted to connect more feedback and teaching to the curriculum, as a resident in primary care wrote: *“[my supervisor] can be more structured about giving feedback and help to achieve the learning objectives”.*

### Assessment

Two categories were allocated to the domain Assessment; e.g., Formal assessment and Planning PGME.

#### Formal assessment and planning PGME

The two categories had the least comments and were only areas for improvement. Residents asked supervisors to do more assessments connected to the curriculum and using the supervision time to structure and follow up the plan for residency. A resident in a department wrote: *“[My supervisor] could be more active in planning my residency with focus on milestones”.*

## Discussion

The aim of this study was to describe the strengths and areas for improvement experienced in the supervision process by residents and supervisors in PGME. Combining the quantitative and qualitative analysis, this study highlights three strengths important to residents and supervisors in the supervision process: the Supervisor as a role model, Being available in time and Giving personal support. Residents learn in workplace by seeing, mirroring, and interacting with support from their supervisors [1, 14]. It is well known that inspiring learning environments are associated with competent role models [30, 31].

The most obvious area for improvement is, Giving and receiving feedback, both positive and constructive, direct connected to daily work or planned structured feedback as, for example, feedback connected to the residency plan. Residents have not been able to answer several feedback questions and supervisors comment they should give more feedback. Supervisors seem to be aware of lack of feedback, yet they struggle to find time och space to be able to give it [32]. Here, residents and supervisor pointed out improved scheduling as an area for improvement. Working together encourage interaction with the possibility to give well-founded feedback in the right time [8]. Supervisors as well as residents also need to understand and embrace their role in giving and receiving feedback [6].

Residents rate their supervisors higher than they do themselves. It is known to be easier to give positive feedback than constructive [33] and residents are also in a dependent position to their supervisors. In a Swedish context, they have the same employee, work together, and many participating supervisors were main supervisors, responsible for assessing the residents. Moreover, the model is new to a Swedish context and supervisors are not used to evaluate themselves, which has been seen in previous studies using self-evaluations [33]. At last, supervisors are struggling to give constructive feedback, afraid of doing wrong or harming the residents [32].

There are differences between the strengths reported in departments and primary care. The supervisor being regarded as an energizer – demonstrating that work is often joyful when supporting medical tasks and taking care of patients – was almost only commented on in departments. The learning environment in departments are varied: in wards, operational theatres, emergency units, and outpatient clinics, and offers more situations where residents and supervisors see each other in work. There is also a clearer progression in medical tasks during PGME in departments compared to primary care, where residents and supervisors take care of similar health problems. In primary care, residents and supervisors meet patients with different diseases and health problems, and they do not always know in advance whether the medical task will be easy or complex task to perform. Residents and supervisors in primary care become experts in taking care of their own patients, which they follow in-person over time, with their problems of varying complexity. While in hospital departments, residents and supervisors take care of more defined medical problems with all the patients they meet.

Residents in primary care highlight the competence of general practice [34]: the principle to work in a patient-centered way, and treat each patient in a balanced and sufficient manner. In primary care, residents work more individually and require guidance in daily work. Surprisingly, supervisors in primary care have not commented about the competence of general practice. Maybe they are practicing more informal teaching, like corridor or ad hoc teaching [35], and not aware of the importance of the general practice competence since the EFFECT questionnaire did not address it.

The strengths reported in this study are in line with previous studies such as role modelling, medically competency, taking good care of patients and giving personal support [4], while the areas for improvement are ingredients incorporated in PGME later according to the switch to outcome-based learning. Supervisors should have pedagogical skills, and know how to give feedback, evaluate and assess residents, and to demonstrate that they need education and support in supervision [5]. Supervisors who get feedback improve their supervision [18] and a safe learning environment includes a feedback-friendly context, where residents are not afraid of being judged [2, 3]. Residents and supervisors in this study point out the need for time and space to give and receive feedback.

Residents have only been able to answer 60% of the questions in domain Feedback, while supervisors have answered most of questions in the same domain. The need of feedback varies during PGME. In the beginning of PGME residents may not have been in all situations mentioned in the EFFECT questionnaire and in the later part of PGME, residents work more independently and do not ask for feedback, there is no long tradition of doing evaluations and assessments. The expectations from residents may differ from the experience of the supervisors. By observing residents and working together, supervisors gain insight into residents’ competence [36, 37] and adapt his or her supervision to help the resident [38]. In this study, not working together, not seeing each other in work and scheduling overall are obstacles for giving and receiving feedback. The low number of evaluations from residents in primary care support this, where the daily work is performed in separate rooms. In departments, residents more often work together with supervisors, but not necessarily with their main supervisor. There is a need for operational managers in departments and health centres to be aware of the importance of scheduling as an instrument to support the learning environment in PGME.

The importance of supervisors as role models in PGME cannot be argued; they form the very basis of the learning environment and are the guarantee for safe and high-quality health care. The findings in this study point out the need of more education in the pedagogical processes in the PGME. Giving and receiving feedback, assessing, using structured feedback and connect feedback to curriculum were all areas for improvement. There is a need of building structures in workplace during PGME, where residents and supervisors have time and space for giving and receiving feedback to increase the performance of residents. In this study, only the EFFECT questionnaire has been in used, while other parts in the EFFECT model, train residents and supervisors to give and receive feedback [39]. The implementation of a feedback model like EFFECT can contribute to developing the supervision process during PGME.

### Strengths and limitations

The study adds to the existing literature about the EFFECT model and its contribution in developing the PGME. The first strength is that the EFFECT model and questionnaire are created through research, incorporating CanMEDS, and have been shown to improve the learning environment as well as promoting giving and receiving feedback between residents and supervisors. Answering the EFFECT questionnaire can be seen as a didactic intervention, because residents and supervisors improve insights of supervision. Secondly, the study combines both quantitative and qualitative methods to deepen the understanding of the supervision process. Thirdly, the research group has increased the credibility of the content analysis by separately coding initially as well as categorising the meaning units. Finally, the study has been done in line with general ethical considerations [28, 29].

There are limitations of this study. Firstly, not all invited supervisors and residents participated and we do not know whether their contribution would have affected the results. The study was conducted in a Swedish context where and may not be completely transferable to PGME in other countries. Another limitation of the study was that the number of evaluations from departments were more numerous, why their opinions might affect the result. Further, because of lack of demographic data in questionnaires, we have not been able to analyse possible differences between residents’ and supervisors’ sex, age or experiences. Finally, a limitation of the result was that giving feedback in smaller units may be more difficult, relating to hierarchies and relations on workplace. Constructive feedback from residents might be undervalued.

## Conclusions and implications

To be able to give feedback, there is a need to see each other in work. Overall, few answers from residents in the primary care, point out the need of creating time and space for residents and supervisors to interact in the workplace. There is also a need to educate supervisors and residents in their two-way role and responsibility in the supervisory process. To increase the opportunities for feedback, scheduling, is an area for improvement. To get rich and well-founded feedback it is important that residents feel safe to give feedback, not afraid of consequences.

The EFFECT questionnaire, used in this study, brought up several aspects of supervision. In primary care, residents pointed out the general practice competence as the most important strength. To make this area more obvious in the future, the EFFECT questionnaire could be complemented with a question about the general practice competence, when used in primary care.

The implementation of a structured feedback model, like the EFFECT, can contribute to improving supervisors and residents experience and competence in giving and receiving feedback. Thereby, improving the learning environment and creating inspiring workplaces. The EFFECT model will be studied further, regarding its contribution in PGME, focusing on the understanding of interactions between residents and supervisors, as well as upward feedback.

## Data Availability

The datasets used and analysed during the current study are available from the corresponding author on reasonable request.
